# Human Positioning in Close-Encounter Photographs and the Effect on Public Perceptions of Zoo Animals

**DOI:** 10.3390/ani12010011

**Published:** 2021-12-21

**Authors:** Meghan N. Shaw, Emily M. McLeod, William T. Borrie, Kelly K. Miller

**Affiliations:** 1 Centre for Integrative Ecology, School of Life and Environmental Sciences, Deakin University, Melbourne, VIC 3125, Australia; b.borrie@deakin.edu.au (W.T.B.); kelly.miller@deakin.edu.au (K.K.M.); 2 Department of Wildlife Conservation and Science, Zoos Victoria, Parkville, VIC 3052, Australia; emcleod@zoo.org

**Keywords:** animal welfare, human-animal interactions, social media, wildlife tourism, attitudes towards zoos, pet ownership, conservation messaging

## Abstract

**Simple Summary:**

Many zoos offer close-encounter experiences, and visitors frequently share photographs from these experiences online. This study measured the effects that viewing such images had on public perceptions of both the zoo and the animals that they saw. Results indicated that viewing different animal species and the different human proximities within these images can affect public perceptions about how zoos care for their animals and views about the use of animals as pets.

**Abstract:**

With the rising popularity of social media, conservation organisations and zoos need to understand its impact on public perceptions of the animals they house and their role in conservation. In addition, many zoos offer close-encounter experiences, and visitors frequently share images from these experiences online. This study measured the effects that viewing such encounter images had on public perceptions of both the zoo and the animals they saw. One of sixteen images was randomly presented to participants in two samples: one of Zoo Community followers and members of Zoos Victoria (*n* = 963), and a representative sample of the Australian public (*n* = 1619). Each image featured one of four animals (Eclectus parrot, Kangaroo Island kangaroo, Monteith’s leaf insect, Centralian carpet python) and one of four human positions (human and animal touching, human and animal ~30 cm apart, human and animal ~1 m apart, animal alone). Results indicated that viewing different animals and the different human positions within these human–animal encounter images can affect public perceptions of zoo animals. In particular, the closer the proximity of a human to an animal in an image, the more likely respondents were to think that the animal was not displaying a natural behaviour and the more likely it was for General Public respondents to think that the animal would make a good pet. These findings can be used by zoos, wildlife tourism, and media organisations to ensure that they are sending clear, positive, and intended messages about zoo facilities and animals, as well as providing insights into animal encounter images in wider settings.

## 1. Introduction

Modern, animal welfare-focused zoos are more than simply places for recreation and entertainment, with many prioritising conservation and education [1,2]. Such zoos are vital for ex situ (such as captive breeding) and in situ (such as wildlife reintroduction) conservation efforts and often implement public education programs to encourage pro-conservation behaviour [3]. As the third-largest funder of conservation globally [4], zoos provide an opportunity for visitors to experience emotional sensitivity towards wildlife [5] as they observe and learn about animals that they would be unlikely to encounter in another setting. Seeing these animals up close can elicit feelings of ‘connectedness’ and kinship, strong conservation attitudes, and pro-environmental behavioural intentions amongst visitors [6].

There is a growing public expectation that zoos care for their animals, both physically and psychologically [4,5]. To ensure public support, and thus funding for conservation efforts, these establishments must develop, achieve, and maintain best-practice standards in animal welfare [7,8]. Visitors are less likely to support a zoo when they perceive an animal in their care to be suffering [8]. Conversely, viewing active animals engaged in natural behaviour can lead to positive visitor experiences and foster conservation learning [9]. Povey and Rios [10] and Packer and Ballantyne [11] argue that, to increase a visitor’s emotional connection to wildlife, visit satisfaction, and pro-conservation behavioural intent, zoos must prioritise and highlight their dedication to animal welfare.

One indicator of good animal welfare is the performance of natural behaviours, defined as “behaviour(s) that animals tend to exhibit under natural conditions, because these behaviours are pleasurable and promote biological functioning” ([8] p. 77). Natural behaviour is often used as an indicator of animal welfare for captive animals as it demonstrates that the restrictions of captivity are not detrimental to the animal [12]. The World Association of Zoos and Aquariums (WAZA), for example, lists natural behaviour as a priority for all members [13].

### 1.1. Close Encounters

The close-encounter experiences commonly offered at zoos [14], which provide an opportunity for visitors to see an animal at close proximity, pose an interesting contradiction for the preference for natural behaviour. These experiences vary depending on the animals and institution involved, but may involve the animal engaging in what could arguably be seen as “unnatural behaviour”—such as feeding from the visitor’s hand, or being held or touched. Although little research thus far has focused on animal welfare during close encounters, some evidence has suggested that human proximity to zoo animals can be a source of enrichment [15,16], and that close observation of zoo animals can be achieved while having neutral or even positive impacts on the welfare of the animal [17,18,19,20,21,22,23]. As suggested by Sherwen and Hemsworth [15], zoos may design their encounters to only feature species whose welfare is not negatively impacted by the practice and that willingly choose to engage in the process. Close encounters also provide an opportunity for zoos to engage with participants to foster a deeper appreciation for wildlife, as well as provide information on how the zoo ensures the welfare of the animal, and of conservation actions that visitors can engage in. Participating in close encounters can lead to visitors reporting greater levels of care and concern for the animal and intentions for further engagement in conservation behaviours [11,24,25]. These encounters will often include a photography opportunity, where visitors may take pictures themselves or have a photo taken with the animal. After their visit, these photos will often be shared on social media platforms.

### 1.2. Social Media

Social media is highly popular and expanding in popularity, providing an effective way to widely share information due to its expansive reach [26]. This phenomenon has been shown to influence a variety of social norms, cultures, and behaviours, with internet terms such as ‘hashtag’, ‘selfie’, and ‘unfriend’ becoming so popular that, in 2014, they were added to the Merriam Webster Dictionary [27]. However, the wide reach and social influence that social media can achieve can also have negative impacts, particularly on wildlife. 

For example, Nekaris et al. [28] describe how many viewers of a YouTube video of a Slow Loris being tickled commented that they thought the animal would make a good pet and was cute, despite the animal’s behaviour being a stress response to its handling. Additionally, YouTube videos with thumbnails featuring humans illegally close to, or even interacting with, Mountain Gorillas were found to not only be more watched than their “legal” counterparts but also growing exponentially in number [29]. Otsuka and Yamakoshi [29] suggest that the social nature of YouTube has increased the perception that tourists are allowed to approach wild animals and even increased the expectation that tourists will have this experience. Therefore, posts featuring humans with wild animals may then allow for misinformation and dangerous behaviours towards animals to be promoted to the public when viewed without appropriate context [30]. Thus, there is a need for further research to explore the impacts that social media may have on people’s attitudes, perceptions, and behaviours towards wildlife. 

Close-encounter images have the potential to attract attention as they contain both novel and emotional components, which are features of the most popular social media content [31,32]. As images target deeper levels of processing than text [33,34,35], they provide a great opportunity to instil information, if the messages received by viewers are those desired by the zoo. Due to their popularity, many close-encounter photographs are posted online, with over 137,000 posts on Instagram with the hashtag #animalselfie and a further 39,600 with the hashtag #closeencounter (as of 10 June 2021). These photos are often posted without any context of the situation they were taken in (e.g., in a zoo setting where the animals are cared for by professionals) or with little to none of the information that was provided to visitors during the experience. Without this context, what messages are the images sending to social media users about the featured animal and the zoo? 

### 1.3. Images of Wildlife

Previous research has highlighted that human–animal photographs can also impact specific viewer attitudes and behavioural intentions. For example, Ross et al. [36] discovered that the presence of a human or humanistic element (e.g., office background, human clothing) in photographs of chimpanzees increased presumptions that the animal was not endangered and that it would make a good pet. Leighty et al. [37] found comparable results when they showed participants images of prosimians (a group of primates including lemurs) in similar contexts. Additionally, viewers of images of wild cats being petted by humans were more likely to express a desire to pet or take a photograph at a wildlife tourism attraction than those who viewed images of wild cats in the wild or against a blank background [38]. These findings are countered by Spooner and Stride [39], who found that zoo visitors who saw images of humans with animals in a zoo context were not likely to desire the animal as a pet, and were more willing to donate to the featured animal’s conservation. However, the mentioned study did find that respondents had more concerns around the featured animal’s welfare when the animal was shown with a keeper or with a member of the public in a zoo setting compared to wildlife tourism and wild settings. 

With these few exceptions, there is limited research on the impact of visual representations of varying species on public perceptions of zoos and animals. Previous research mostly focuses on primates, though zoos host many species, and the animals used for close encounters vary across taxa [14]. Moreover, there is little research on the impact of viewing photo elements, such as a human’s proximity to, and interaction with, the featured animals in photographs, particularly within a zoo setting. 

This study aimed to determine the impact of varying human proximities to animals of different taxa in close-encounter images on viewers’ perceptions of the zoo animal in the photograph. Perceptions are defined as “a thought, belief, or opinion, often…based on appearances” [40] and have historically been thought to determine attitudes along with a person’s feelings and reactions [41]. 

We hypothesised that respondents would be less likely to agree that the animal they saw was cared for by the zoo or displayed a natural behaviour when it was shown close to a human. This reflects the results of Spooner and Stride [39], where animal welfare was seen as a concern by viewers of animal-human imagery within a zoo context, as well as other studies of zoo animals that show that species do alter their behaviour in the presence of humans [42]. We also hypothesised that respondents would be more likely to agree that the animal they saw was not endangered in the wild and would make a good pet when shown close to a human, reflecting the results of Ross, Vreeman, and Lonsdorf [36] and Leighty et al. [37].

## 2. Materials and Methods

Human ethics approval was granted for this project by Deakin University’s Human Ethics Advisory Group, under the reference STEC-65-2019-SHAW. The study was exempt from animal ethics approval as the animal photography followed the strict protocol of the zoo for animal encounters.

### 2.1. Study Design

To investigate the impact of photographs on perceptions of zoos and animals, sixteen photographs were taken across two days at Zoos Victoria properties—Melbourne Zoo and Healesville Sanctuary—with full permission and assistance from the organisation. The four animals involved in the photos were already familiar and comfortable with human proximity and were closely monitored by zoo staff during the photography session, each of which lasted for less than 5 min. Each of the sixteen photographs featured one of four human positions and one of four Featured Animals. The lighting, human participant, and clothing were kept constant between all images. For each animal, the background was also kept constant. The human model within the photographs was a volunteer. The photos were taken in the manner and style commonly seen in close-encounter photos to create images that accurately represented these scenarios.

### 2.2. Selected Featured Animals

**Figure 1:**
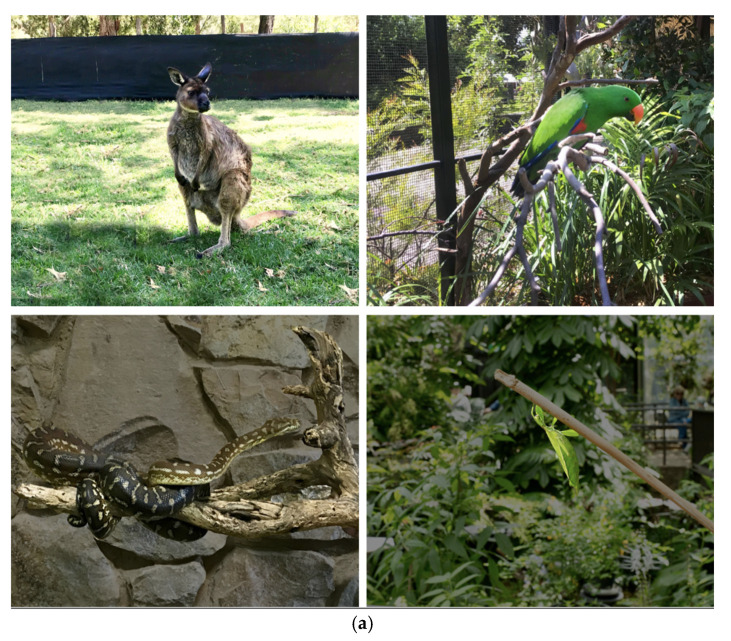

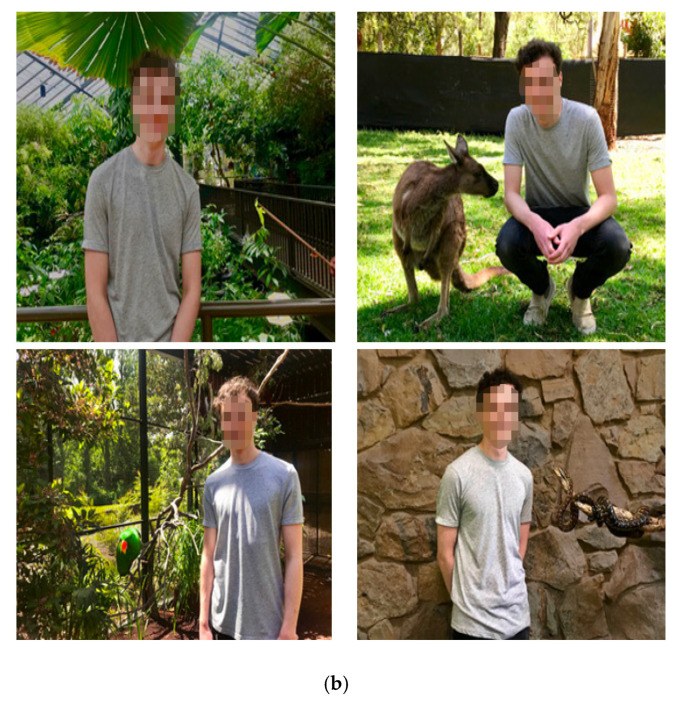
(**a**) The four Featured Animals, photographed with no human present, and (**b**) the four Featured Animals, photographed with the human ~30 cm from the animal.

The four animals selected (hereafter referred to as “Featured Animals”) were selected with two criteria: (i) the species were native to Australia, (ii) the species were representative of the taxa commonly included in close encounters. Australian species were chosen as the respondents in the samples were Australian, and thus these species were likely to be animals that the respondents would see in their local zoos. The four species, as shown in Figure 1, were from different classes: mammal—the Kangaroo Island kangaroo (*Macropus fuliginosus*), bird—a male Eclectus parrot (*Eclectus roratis*), reptile—a Centralian carpet python (*Morelia bredli*), and insect—a female Monteith’s leaf insect (*Phyllium monteithi*). 

### 2.3. Human Position

The four human positions (hereafter referred to as “Human Positions”) reflect those common in close-encounter images, ranging from touching to no human presence (see Table 1). This variable was designed to measure the effect of a human’s position in the photograph, and, as such, the same model was used across all photos to control for any effect that the appearance of the person may have had on how the photo was perceived. We chose to include both a 30 cm and 1 m condition as, to our knowledge, the role of distance between a human and an animal in images on public perceptions has not yet been investigated. The role of human–animal proximity has been under scrutiny in recent years, with many studies finding that wild animals can experience negative welfare when in close proximity to humans [42]. Because of this, many reserves and shelters now have regulated distances for viewing wildlife [43]. As the Featured Animals in this study were relatively small, the distances of 30 cm and 1 m were selected so that the animal would be visible within the photograph. This also more closely relates to the close-encounter setting and could provide insight into whether altering the distance of a human from an animal in close-encounter imagery is discernible and whether it impacts perceptions gained from viewing these images. 

### 2.4. Survey Design

Both the survey layout and questions were designed for clarity and consistency of interpretation. Pre-tests were conducted with a small group of 20 university students with various levels of familiarity with zoos, to ensure sufficient variation in responses and to revise any unclear or ambiguous questions or instructions [44].

The survey included (in order):

- A link to a Plain Language Statement that outlined the research, participant rights, and ethics approval in more detail.
- One photograph presented per participant depicting one Featured Animal and one Human Position (for all images, see Supplementary Figure S1). The photographs were equally but randomly assigned to participants to ensure comparable sample sizes. The choice to assign only one photograph was made to reflect the work of Ross, Vreeman, and Lonsdorf [36] and Leighty et al. [37] and to remove the impact of order effects. Respondents were not informed as to which zoo these images were taken at, nor were they aware that other participants potentially saw a different image.
- A set of agreement statements measuring perceptions of zoos and the animals in the photographs:
  1. The animal is cared for by the zoo;
  2. The animal is displaying a natural behaviour;
  3. The animal would make a good pet.

These statements were chosen as they measured public perceptions of the zoo’s care for the animals, both as an outright question and through a partial measure of animal welfare, or were consistent with previously established work on public perceptions of animals in photographs [36,37]. The level of agreement with these statements was measured through a 7-point Likert-type scale that ranged from 1 = strongly disagree to 7 = strongly agree, with the midpoint of 4 = neither agree nor disagree.

- A set of attitude scales measuring respondents’ attitudes towards wildlife and zoos. These questions form part of a different study and the results will be reported elsewhere.
- A set of demographic questions (gender, age, level of education, residential location, zoo membership status, zoo visitation regularity, and conservation organisation membership status) to allow for the description of sample characteristics.

At the end of the survey, participants were offered the opportunity to enter a draw for three AUD 50 gift cards. As a token of gratitude, participants were also given access to seven downloadable desktop backgrounds. The average response time for the survey was approximately 12 min.

### 2.5. Sample Recruitment

The study encompassed two different audiences:

1. Zoo members or zoo social media followers (hereafter referred to as the Zoo Community sample), who may be more likely to post and/or see photos of close encounters. This audience is likely to be more exposed to the previous work of zoos in both welfare and conservation efforts than the general public [45], as they have made the conscious choice to receive promotional and educational materials from the zoo across their social media platforms, and thus are likely to hold an interest in zoos and animals. This sample may access such knowledge when forming attitudes based on the images they see, such as seen in Cohen [46].
2. The Australian general public, members of which may also see these posts on social media, but may not be as familiar with zoos and wildlife (hereafter referred to as the General Public sample).

The two populations consisted of different audiences, who may thus respond in different ways. Thus, samples for each of these populations were recruited in different ways, to reflect the variation in these groups.

The Zoo Community sample consisted mostly of “followers” of zoo and Australian conservation organisations across Facebook, Twitter, Instagram, LinkedIn, and Reddit. A post containing a link to the online survey was shared 20 times (staggered, every two days) on the social media accounts of Zoos Victoria, researchers, and conservation groups (e.g., Field Naturalists of Victoria). Most of these groups encompass Australian residents only.

The General Public sample, selected to be demographically similar to the Australian public in age, gender, and urban/rural locality, was recruited from panel members of the Online Research Unit (comprising a list of 350,000 individuals) [47]. Panel members were directly emailed an invitation to participate, and recruitment continued until representative quotas of respondents were met. Surveys with no data, such as those who had only accessed the first page, or whose response time was under 2 min (as it was determined through the pre-test that the survey took at least 5 min to complete), were removed from analyses. Respondents who indicated that they were from outside Australia were later removed from analyses. 

### 2.6. Analysis

The overall survey data were non-normally distributed, as indicated by Kolmogorov–Smirnov [48] and Shapiro–Wilk [49] testing, so we decided to analyse the impact of Human Position, Featured Animals, and other demographics on each agreement statement with a linear regression approach. 

Following the approach of Van der Meer, Botman, and Eckhardt [38], we collapsed the 7-point Likert scale categories into 5 for each of the agreement statements, to allow for an adequate number of events for each variable. Thus, “strongly disagree” and “disagree” were combined, as were “strongly agree” and “agree”. 

Pearson’s chi-square tests were then conducted to compare the two samples across each of the demographic questions. The results showed significant differences between the two samples for most demographic questions, so the samples were separated for further analyses. A principal components analysis was conducted of the three agreement statements for both samples, to determine if the agreement statements were measuring different perceptions. Results indicated that none of the three agreement statements were closely correlated with each other for either sample, so distinct statistical analysis was appropriate. 

As the responses to each of the agreement statements represented a scale of agreement, we fitted ordinal regression models to each of the three statements for each sample, as done by Van der Meer, Botman, and Eckhardt [38], for a total of 6 models. As demographic information such as gender, age, and education level has been shown to impact our perceptions and attitudes towards animals [50,51], all collected demographic information, including zoo visitation and zoo membership, was added to the original models, as well as the Human Position and Featured Animal that each respondent saw in their allocated image. All possible interactions between each of these variables were also added. Ordinal regression modelling was conducted with SPSS software version 25.0 [52] using forward selection and then a GENLIN procedure, which is centred on likelihood testing. An ordinal regression was not suitable for the second model that examined the relationship between the variables on agreement that the zoo cares for the featured animal for the General Public (Pearson’s goodness of fit test *p* < 0.05, test of parallel lines *p* < 0.05). Instead, a multinomial regression was fitted. For all models, any variables and interaction terms that did not have a significance level (*p*-value) of > 0.05 were removed via forward selection (for a full list of variables tested, see Supplementary File 1, and any non-significant interaction effects). For comparison analyses, the Kangaroo was set as the reference animal as it was the most recognised species and least likely to be a pet. The animal-alone condition was also set as a reference category for comparison of analyses of Human Position effects.

For both samples the model fit to the statement “the featured animal is cared for by the zoo” and for the Zoo Community samples’ model fit to the statement “the featured animal would make a good pet” included a significant interaction term between the Featured Animal and the Human Position, so post-hoc Pearson’s chi-square testing with Bonferroni corrections were conducted to determine the nature of these interactions. 

## 3. Results

A total of 2582 surveys were completed (Zoo Community sample, *n* = 963; General Public sample, *n* = 1619). There was a difference between the two samples by most demographics, including gender identity X^2^ (1, *n* = 2450 = 275 *p* < 0.001), age (H = 160.42, df = 1, *p* < 0.001), education (H = 29.44, df = 1, *p* < 0.001), zoo membership X^2^ (1, *n* = 2471 = 461, *p* < 0.001), frequency of zoo visitation (H = 327.72, df = 1, *p* < 0.001), and conservation organisation membership X^2^ (1, *n* = 2473 = 159, *p* < 0.001). 

### 3.1. Demographics

#### 3.1.1. Zoo Community

Approximately 60 respondents were allocated to each of the 16 images in the Zoo Community sample. Across the images, a maximum of 91.5% and a minimum of 68.4% of those allocated to each image were female, mean age varied from 39.12 ± 1.9 (mean ± SE) to 43.46 ± 2.1 (mean ± SE), and education level ranged from 9.89 ± 0.39 (mean ± SE) to 11.15 ± 0.368 (mean ± SE), which is the equivalent of an Australian Advanced Diploma (AQF Level 6) for the first and a Bachelor’s degree (AQF Level 8) for the second. These variations in demographics between the populations who saw each image were due to the random allocations of the Qualtrics software. 

#### 3.1.2. General Public

Approximately 101 respondents were allocated to each of the 16 images in the General Public sample. Across the images, a maximum of 58.4% and a minimum of 41.6% of respondents allocated to each image were female, mean age varied from 47.38 ± 1.6 (mean ± SE) to 53.4 ± 1.7 (mean ± SE), and education level ranged from 9.24 ± 0.38 (mean ± SE) to 10.36 ± 0.30 (mean ± SE), which are the equivalent of an Australian Diploma (AQF Level 5) for the first and a Bachelor’s degree (AQF Level 8) for the second.

Table 2 highlights the demographic characteristics for the Zoo Community sample, which are different from the 2016 Australian Demographic Statistics [53], particularly for gender and education. Females were heavily represented in the Zoo Community sample, which was also skewed towards university-educated respondents. However, the sample was similar in age distribution to Australian Facebook users [54]. The General Public sample broadly reflected the demographic profile of the Australian public, although it, too, differed in education level. Importantly, for both samples, each of the demographic variables was found to have no relationship with responses to each of the three agreement statements.

### 3.2. The Animal Is Cared for by the Zoo

#### 3.2.1. Zoo Community

Overall, 86.5% of respondents agreed to at least some degree that the animal they saw was cared for by the zoo. Within the model, an interaction between the Featured Animal and the Human Position was found to impact the level of agreement with this statement (X^2^ = 17.32, df = 9, *p* = 0.04). 

**Figure 2:**
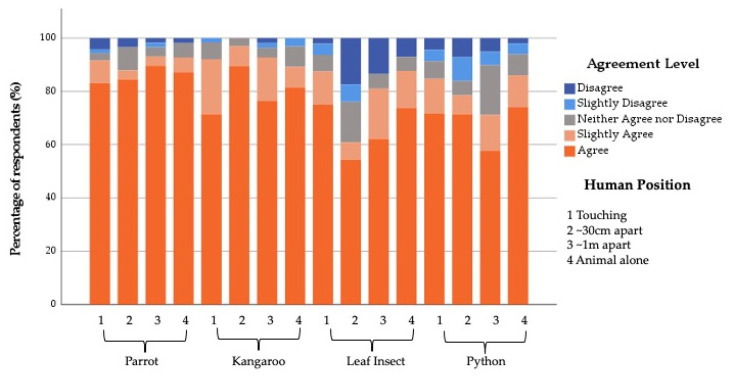
Percentage of Zoo Community respondents’ agreement with the statement “the featured animal is cared for by the zoo” in relation to the allocated images (Featured Animal by Human Position).

A post hoc chi-square analysis showed that a higher percentage of people disagreed that the animal they saw was cared for by the zoo when they saw the leaf insect at a distance to the human (~30 cm and ~1 m apart) and the snake ~1 m from the human (X^2^ = 101.829, df = 45, *p* ≤ 0.001) (Figure 2). 

#### 3.2.2. General Public

**Figure 3:**
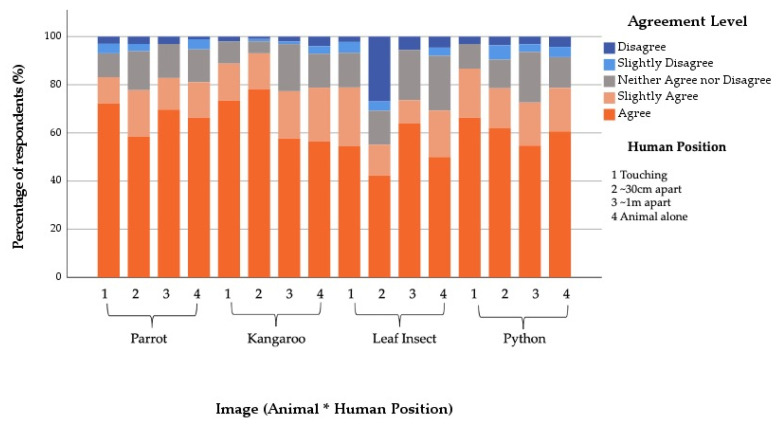
Percentage of General Public respondents’ agreement to the statement “the featured animal is cared for by the zoo” in relation to the allocated images (Featured Animal by Human Position).

Overall, 79% of respondents in the General Public sample agreed to at least some degree that the animal they saw was cared for by the zoo. A multinomial regression indicated that there was a significant interaction term that impacted the level of agreement with this statement (X^2^ = 62.91, df = 36, *p* = 0.04), with those who saw the Kangaroo ~30 cm away from the human having a slightly higher percentage of agreement with the statement. Those who saw the Leaf Insect ~30 cm away from the human had a much lower level of agreement with the statement (X^2^ = 136.096, df = 45, *p* < 0.001) (Figure 3). 

### 3.3. The Animal Is Displaying a Natural Behaviour

#### 3.3.1. Zoo Community

**Figure 4:**
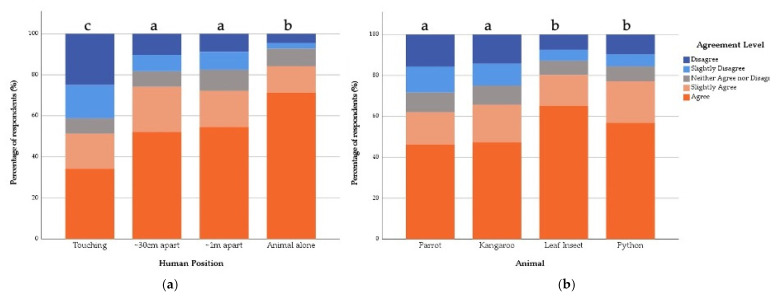
Percentage of Zoo Community respondents’ agreement with the statement “the featured animal is displaying a natural behaviour” in relation to the (**a**) Human Position shown and (**b**) Featured Animal shown. Same letters indicate no statistical difference and different letters indicate statistical differences between variables in the fitted model.

Overall, 70.6% of respondents agreed to some degree that the animal they saw was displaying a natural behaviour. There was no significant interaction effect (X^2^ = 9.31, df = 9, *p* = 0.409). However, Human Position (X^2^ = 71.71, df = 3, *p* ≤ 0.001) and Featured Animal (X^2^ = 21.55, df = 3, *p* ≤ 0.001) both had an impact on the levels of agreement with this statement. Compared to the control image, where the animals were shown alone, those who saw the animal being touched by a human were 0.186 times less likely to agree that the animal was displaying a natural behaviour (Table 3). A similar pattern occurred for the other positions, with those who saw the animal at ~30 cm away from the human 0.47 times and those who saw the animal at a ~1 m distance 0.5 times less likely to agree that the animal was displaying a natural behaviour (Table 3 and Figure 4). 

Compared to the Kangaroo, those who saw the Python were 1.55 times and those who saw the Stick Insect were 2.17 times more likely to agree that they were displaying a natural behaviour (Table 3 and Figure 4). 

#### 3.3.2. General Public

**Figure 5:**
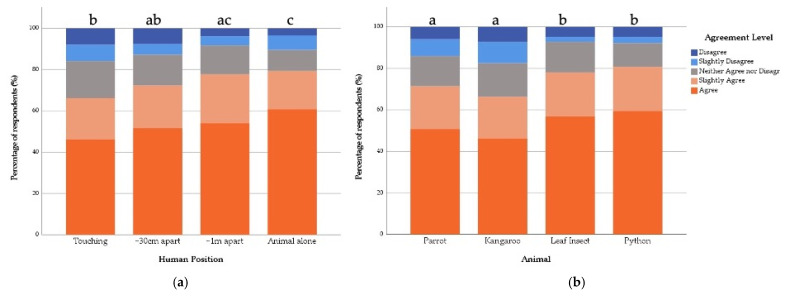
Percentage of General Public respondents’ agreement with the statement “the featured animal is displaying a natural behaviour” in relation to the (**a**) Human Position shown and (**b**) Featured Animal shown. Same letters indicate no statistical difference and different letters indicate statistical differences between variables in the fitted model.

Overall, 73.9% of respondents in the General Public sample agreed to some level that the animal they saw was displaying a natural behaviour. Again, there was no significant interaction term in the fitted model (X^2^ = 5.81, df = 9, *p* = 0.76), but Human Position X^2^ = 20.90, df = 3, *p* ≤ 0.001) and Featured Animal (X^2^ = 24.09, df = 3, *p* ≤ 0.001) both had significant main effects. Compared to the control images, where the animals were shown alone, those who saw the human touching the animal were 0.54 times and those who saw the animal and human ~30 cm apart were 0.47 times less likely to agree that the animal was displaying a natural behaviour (Table 4 and Figure 5). Compared to the Kangaroo, those who saw the Python were 1.85 times and those who saw the Stick Insect were 1.66 times less likely to agree that the animal was displaying a natural behaviour (Table 4 and Figure 5).

### 3.4. The Animal Would Make a Good Pet

#### 3.4.1. Zoo Community

**Figure 6:**
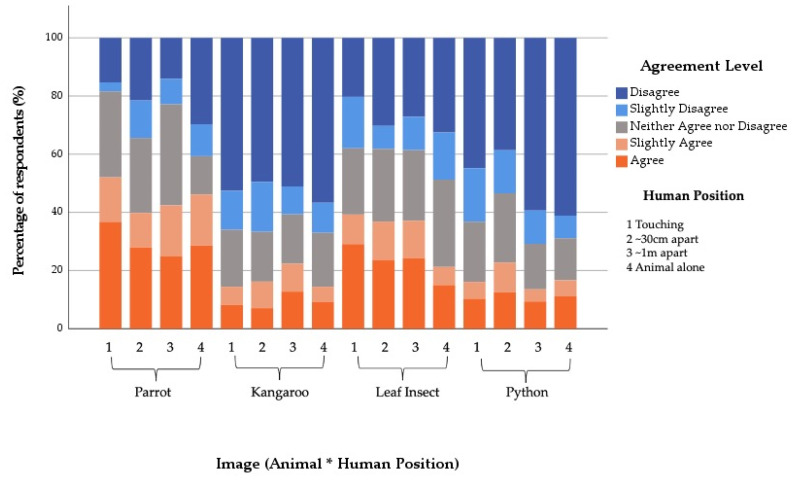
Percentage of Zoo Community respondents’ agreement with the statement “the featured animal would make a good pet” in relation to the allocated images (Featured Animal by Human Position).

Overall, 16% of Zoo Community respondents agreed to some level that the animal they saw would make a good pet. The fitted model included an interaction term (X^2^ = 19.86, df = 9, *p* = 0.019). A chi-square analysis (X^2^ = 125.824, df = 60, *p* ≤ 0.001) showed that although most people who saw each image disagreed with the statement, a high proportion of those who saw the Parrot with a human (touching, 30 cm or ~1 m apart) and the Leaf Insect ~30 cm apart or without a human did agree with the statement to some level. Additionally, the groups who saw the Kangaroo ~30 m apart or without a human or the snake ~1 m from a human expressed lower agreement that the animal would make a good pet (Figure 6). 

#### 3.4.2. General Public

**Figure 7:**
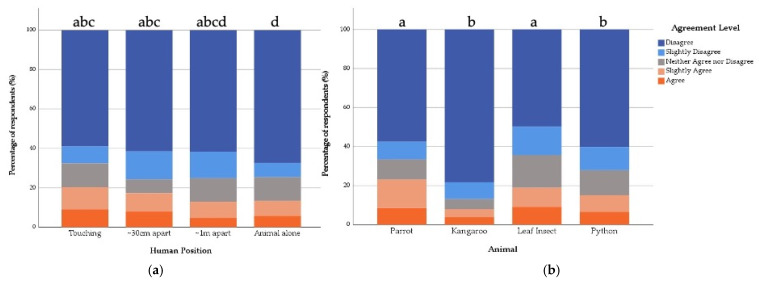
Percentage of General Public respondents’ agreement with the statement “the featured animal would make a good pet” in relation to the (**a**) Human Position shown and (**b**) Featured Animal shown. Same letters indicate no statistical difference and different letters indicate statistical differences between variables in the fitted model.

Overall, 27.9% of respondents from the General Public sample agreed with the statement “the animal would make a good pet” to some level. The fitted model did not include a significant interaction term (X^2^ = 11.88, df = 9 *p* = 0.220), but both Human Position (X^2^ = 11.41, df = 3, *p* = 0.01) and Featured Animal (X^2^ = 162.83, df = 3, *p* ≤ 0.001) were found to have an impact on respondents’ level of agreement with this statement. In comparison to the control images of each of the animals alone, those who saw the animal and the human at ~1 m apart were 1.29 times, those who saw the animal and human ~30 cm apart were 1.42 times, and those who saw the human and the animal touching were 1.56 times more likely to agree that the animal they saw would make a good pet (Table 5 and Figure 7). In comparison to the Kangaroo, those who saw the Parrot were 4.32 times and those who saw the stick insect were 2.79 times more likely to agree that these animals would make a good pet (Table 5 and Figure 7).

## 4. Discussion

This study highlights that the perceptions drawn from viewing close-encounter images can differ based on the animal featured, the human’s presence and position in the frame, and the audience viewing the photograph. In particular, the Zoo Community agreed that the animals they saw in such photographs were cared for by the zoo 7.5% more than members of the General Public. For both samples, the presence of a human, and a human’s proximity to a featured animal, lowered agreement that the animal was displaying a natural behaviour. In addition, the Parrot and Kangaroo were less likely to be considered as displaying natural behaviour in close-encounter photographs in comparison to the Python. Finally, when considering whether the Featured Animal would make a good pet, almost one third of the General Public stated that they agreed. The presence of a human in these images, as well as the Featured Animal species, also impacted agreement with this statement. These results somewhat reflect our hypotheses but emphasise that the impact of image features on different audience perceptions is a more complex relationship than anticipated. 

These findings, as discussed in more detail below, may provide insight into how zoos and other wildlife organisations can best design their close-encounter experiences and deliver more precise messaging regarding their animal welfare and wildlife conservation priorities. Zoos providing close encounters typically determine whether photos can be taken, who takes them, and how elements are positioned within the photo frame (specifically, human presence, interaction with and distance to encounter animals in photographs, what species are involved, and the nature of the backdrop provided). Although zoos do not have control over the images that visitors post to social media, an understanding of how these images influence viewer perceptions is important in framing clear messages. Indeed, this study posits that the conditions of the close encounter have an important influence, specifically on the viewers’ perceptions of animal welfare and wildlife conservation.

### 4.1. The Animal Is Cared for by the Zoo

Although the pattern of agreement for this statement was mixed in both samples, highlighted by the significant interaction term between Featured Animal and Human Position, there was a key difference in the overall levels of agreement between the Zoo Community and General Public samples, with 7.5% more of the Zoo Community sample agreeing that the animal they saw was cared for by the zoo than was the case with the General Public sample. 

Zoo visits can help to facilitate a connection to animals [55] and are associated with greater levels of understanding about biodiversity and pro-environmental actions [56]. As the Zoo Community sample had higher levels of zoo visitation and zoo/conservation organisation membership than the General Public sample (Table 2), these respondents may have had greater knowledge of how modern zoos ensure the welfare of the animals in their care and of their conservation work, or they may simply be more supportive of zoos in general. 

With the Zoo Community sample’s demographic similarity to Australian Facebook users, they may represent the subset of the population most likely to take encounter photos and share them on social media. They are also potentially the most likely to engage with zoo- and conservation-related social media content and, thus, be the first impacted by viewing such images. Hence, zoos and such organisations should continue to pay attention to this audience and how they are impacted by close-encounter images. 

By comparison, the General Public sample may have less knowledge of zoos and animals than the Zoo Community sample. Although this group may not be first to engage with zoo social media content, social media allows people to post or share any content, and, thus, it is likely that both close-encounter images posted by visitors and content posted by a zoo or aquarium and shared by their followers will reach a more general audience than merely those who follow zoo or aquariums’ social media. Indeed, even a study involving zoo visitors found that animal welfare was considered more of a concern when viewing zoo imagery than wild imagery [39], and these concerns are likely further exacerbated in non-zoo audiences. Without direct knowledge of zoos and animals, the messages that the general public may receive about zoo practices may differ from what was originally intended by the post and negatively impact a zoo’s social licence.

### 4.2. The Animal Is Displaying a Natural Behaviour

There was a high level of consensus among each sample’s pattern of agreement with the statement “the animal is displaying a natural behaviour”, and these were also impacted by both the Human Position and the Featured Animal. 

Indeed, for the General Public, both the touching and ~30 cm conditions resulted in significantly lower levels of agreement than the “alone” condition, whereas a human at a distance of ~1 m did not.

This pattern in results may have occurred due to a perception that an animal, particularly a non-domesticated species, may be less likely to behave naturally when a human is present and close to an animal. This is similar to the results of Spooner and Stride [39], who found that animal welfare was seen as a concern by viewers of animal-human imagery within a zoo context, and perhaps it was similar concerns that played a role in respondents’ perceptions of natural behaviour here. Furthermore, studies of zoo animals show that species do alter their behaviour in the presence of humans and that negative effects seem to occur more frequently when a zoo visitor is interacting inappropriately with the animal or the animal is unable to escape the interaction [43]. Some viewers may have knowledge or perceptions around such animal behavioural changes, which may guide their responses. Viewers may believe that encounter images are engineered, unnatural, and that the animal is under human control, believing that the animal is unable to escape the interaction. Additionally, viewers may be subconsciously influenced by the height difference between the human and animal. In film semiotics, height is used to convey power; thus, a greater height of the human over the animal could imply that the human has dominance and power over it [57], which in turn would lead to the animal being unable to act naturally. 

However, we must also consider what respondents believe “natural behaviour” is, and how these beliefs may guide responses. Research has shown that humans tend to misinterpret animal behaviour as we may base our understanding of animal behaviour on popular media, which often shows wild animal behaviour as active and free [58,59,60], and captive animal behaviour as stressed and stereotypic, such as in Blackfish [61]. This highlights that natural behaviour may likely be seen as the active behaviours of hunting, mating, and playing, as shown in wildlife documentaries, and that the behaviour shown by zoo animals is unnatural and a result of their “limited welfare”. Furthermore, this argument is commonly reflected by animal welfare advocates [62]; thus, these results highlight that encounter images are sending negative animal welfare messages. Further research should determine how perceptions of “natural behaviour” play into people’s understandings of animal welfare. 

It is interesting to note that images showing a human at a slightly further distance of ~1 m from an animal do not receive significantly different agreement scores in the General Public sample to images showing an animal alone, but images showing humans at ~30 cm or touching the animal do. This could suggest that there is the perception amongst non-zoo followers that keeping one’s distance, even at only 1 m, from animals does not impact animal behaviour or welfare. Such findings are intriguing as they suggest that there may be certain distances that the general public believe they can keep from wildlife that will ensure the animal’s welfare, and that this may even be such a small distance as ~1 m. Future research could determine at what precise distances these perceptions change for different taxa, and how receptive the general public are to keeping their distance from wildlife in similar tourism experiences. 

Levels of agreement that the animal featured was displaying a natural behaviour also differed based on which animals were featured in the image. Compared to the baseline species (the Kangaroo), the Leaf Insect and the Python were less likely to be considered as displaying a natural behaviour. Previous literature may provide some insight into the underlying perceptions guiding this trend in responses, highlighting that people tend to attribute more cognitive ability to bird and mammal species [63] and even prefer them to other species [64]. It is suggested that this is due to their relative similarity in behaviour and appearance to humans [65,66,67]. Thus, people may report stronger feelings of identification with, and empathy for, birds and mammals in comparison to insects and reptiles, which may, in turn, lead to a belief that they understand the behavioural and emotional needs of these species [64,65,66,67]. These feelings of empathy could then lead to a lower level of agreement that mammals and birds are displaying natural behaviours due to the restrictions of captivity, which is not seen as strongly for the other species. We argue that these results further support the fact that respondents may see “natural behaviour” as an indicator of animal welfare. 

Additionally, respondents may be influenced by their previous experiences with reptiles and insects, particularly in the media. Representations of reptiles and invertebrates are lacking in comparison to more “charismatic species” [68], which may also explain why understandings of such species are limited, and thus why levels of agreement are lower. Moreover, many studies show that there is an underlying fear of some reptiles and invertebrates within communities, with perceptions that such species are pests, dangerous, dirty, and unable to be controlled [69,70], and even that they do not have active consciousness and thus do not perform active behaviours [63,69]. Future studies could test the theory of prior species knowledge and feelings of affiliation on perceptions of animal behaviour, to determine how strong these impacts are on understandings of various species. We also note that feelings of ambivalence towards the featured species may have impacted how viewers responded. 

### 4.3. The Animal Would Make a Good Pet

Perhaps the most concerning finding of this study is that nearly one third of General Public respondents agreed that the animal they saw in a close-encounter photograph would make a good pet. This result is similar to the findings of Ross, Vreeman, and Lonsdorf [36] and Leighty et al. [37] that viewing human–animal imagery increases the appeal of the ownership of wildlife, but somewhat refutes the findings of Spooner and Stride [39], who found that zoo-based human–animal imagery did not increase desires to own the animal in the image. As Spooner and Stride [39] conducted their research on zoo visitors only, and our results were seen only in our General Public sample, we suggest that this provides evidence that the audience and their connection to the zoo may impact their response to this statement. This is reflected in the fact that 16% of the Zoo Community sample agreed with this statement, compared to 27.9% of the General Public. We also note that some respondents stated that they were unable to see the Leaf Insect properly in the ~1 m and ~30 cm Human Positions, which may explain some of these mixed results for the Zoo Community sample. 

For the General Public sample, the level of agreement that the animal they saw would make a good pet was further impacted by the presence of a human within the image. Indeed, not only was a “touch” position more likely to increase this perception compared to the animal alone, but also positions where the human was at ~30 cm and ~1 m from the Featured Animal, reflecting the findings of Ross, Vreeman, and Lonsdorf [36]. However, these findings add further depth to this previous work, suggesting that although human presence in human–animal images does increase perceptions that the Featured Animal would make a good pet, the strength of this influence may also be impacted by the distance of the human from the animal in the photo, as shown in Figure 7.

We suggest that the presence and distance of a human from an animal in such images may play a strong role in perceptions of ownership or companionship with the Featured Animal, either by highlighting the dominance of humans over wildlife or by increasing perceptions that such behaviour is appropriate. Both suggestions are supported by Siriwat and Nijman [71], Otsuka and Yamakoshi [29], Van der Meer, Bockhart, and Eckman [38], and Stride [39], who have all found that imagery of humans and animals together on social media increases expectations that viewers can approach, touch, and own animals that are not considered domestic. As such images increase in popularity and number, the theories of social norms [42] and repeat messaging [72] suggest that these behaviours may increase in number and severity, as seen by Otsuka and Yamakoshi [29]. Thus, images with humans and animals together should be used with caution, as they may model the idea that owning illegal exotic wildlife is appropriate. We do note, though, that respondents are reporting a perception, not an actual behaviour, and this does not necessarily mean that all respondents would then seek such animals as pets. 

The featured species within these images also impacted the level of agreement that the General Public showed with this statement, which suggests that our perceptions of what makes a “good pet” are also impacted by the species of animal. For example, compared to the Python, respondents were more likely to agree that the Parrot would make a good pet. Although we did not assess prior experience and knowledge of exotic pets, different species of Parrot are a common choice of pet worldwide, including the Eclectus Parrot [73]. However, the Python is also a common exotic pet worldwide but did not receive a high level of agreement in this study, although this could be due to the commonly held fear of snakes, and the view that they are dangerous [70]. The Leaf Insect also received higher agreement than the snake regarding this statement. Again, this perception may be driven by the view that invertebrates are “mindless” and thus are easier to control and take care of [69], with fewer welfare implications from their ownership. 

The Kangaroo did not receive significantly different levels of agreement compared to the snake, which could suggest that charismatic megafauna (large mammals with high public popularity) are simply less likely to be considered good pets. This theory is further supported by Van der Meer, Eckman, and Bockhart [38], who saw similar trends when showing images of humans and big cats, and Spooner and Stride [39], who suggest similar findings with images of large mammals. Again, this may be due to the increased emotional connection that humans seem to have with large mammals, which may foster the belief that such animals belong in their natural habitat to increase their welfare and happiness, and thus should not be owned [63,74]. Alternatively, this may be due to a perceived difficulty of the logistics of keeping such large animals in the home; as we did not measure respondents’ knowledge of or feeling of connection to these species, we suggest that they should be explored in future research to determine what makes a wild animal more likely to be perceived as a “good pet”. 

### 4.4. Limitations and Future Research

This study should be considered an initial exploration of the role of animal photographs in public perceptions, with significant potential for further research. Although there were significant differences in perceptions, these were often small differences. It would be worthwhile for future researchers to investigate respondents’ opinions of zoos and wildlife conservation organisations and how these impact responses to viewing these images. Researchers could also determine which of the images used in this study viewers find socially acceptable and whether they have participated in similar photographs themselves. Furthermore, it is noted that the animals used in this study cannot accurately represent all other species within their taxa, with species varying across an endless array of variables. Future research could also consider the inclusion of different species to determine the impact of viewing native versus non-native species.

Additionally, the agreement statements used were exploratory in nature and thus were subject to various interpretations by different respondents. We acknowledge that a variation in understandings of these statements may have impacted how respondents scored their level of agreement. The development of multiple items for each dependent variable would likely allow greater precision.

It should also be noted that these results could vary depending upon the human model used. The images included in this work featured only one human, a young, Caucasian male, as have previous studies in a similar area [36], including recent work on the use of celebrities for conservation campaigns [75]. Further work should aim to reflect the diversity of people using social media, and thus could explore the impact of different human models in conservation and animal imagery, varying by gender, ethnicity, age, ability, and role in the community. 

As perceptions of different species tend to vary with culture and age [76,77], cross-cultural, multinational, and cross-generational studies could also be conducted to understand these effects in more detail, and to determine how messaging efforts may differ internationally. Additionally, as described in the demographic overview, these samples are not entirely representative of the general public and all social media users. Previous participant knowledge of zoos and animals was unknown. Future research could determine the impact of viewing similar photos on audiences with clearly defined levels of knowledge towards zoos and the featured animals. 

Lastly, natural behaviour is not the only criterion that can be used to determine animal welfare and, thus, a more detailed study including other measures may provide further insight into how photos and images can be used to best support the conservation objectives of the modern zoo.

### 4.5. Implications

Viewing human–animal close-encounter images impacts viewer perceptions of the zoo and their animals. However, participating in an encounter can provide a unique experience to zoo visitors and help to foster a sense of connection to wildlife [78]. Additionally, strengthening of pro-conservation beliefs gained during the experience may occur when visitors view memorabilia such as images of their experience and repeat messaging [79,80]. Thus, to promote certain perceptions most effectively, zoos must carefully manage their media to ensure that they are effective in achieving their specific goals. Part of this should involve considering the audiences that will see the media and how they might engage with it. Any unintended effects such as eliciting a negative view of animal welfare or encouraging illegal or inappropriate pet ownership should be considered, particularly as audience perceptions may be impacted by outside sources such as television, news, and other social media posts that are not under the control of the zoo. Thus, those offering close-encounter experiences should consider how they create photographic opportunities for visitors that then result in memorable images, which are likely to be shared on social media. Zoos could develop specific messaging to educate visitors and participants in animal encounter experiences as to the best way to share their photographs online to encourage zoo support and pro-conservation behaviour, such as asking participants to only share images of themselves not touching the animal, or suggesting that text is included in social media posts that sends a conservation message. Zoos could also choose to follow the recently developed Best-Practice Guidelines for Responsible Images of Non-Human Primates, as published by the IUCN/SSN specialist group [81], and could apply these principles to images of animals of differing taxa to ensure that they are sending the most responsible messages about themselves and the animals they advocate for.

## 5. Conclusions

This study has shown that viewing images of humans and animals together impacts perceptions of both the zoo and the animals seen. As such, this study informs our understanding of the possible outcomes associated with viewing images and highlights the importance of understanding the audiences who may see these images. The findings have important implications for how zoos and aquariums share imagery online in a way that promotes positive perceptions of wildlife and of how such organisations care for their animals.

## Figures and Tables

**Table 1:** Different Human Positions and the purpose of their inclusion in the study.

Human Position	Description	Purpose
Animal alone	The animal by itself, without human presence	A “control” to assess the difference in participant attitudes to photographs with and without human presence
Touching	A visible touch of the animal by the human model	To measure the impact of viewing an interaction between the human and the animal
~30 cm apart	The same positioning as for touching, but without the touch interaction	To measure the impact of the human’s distance from the animal
~1 m apart	The human and the animal positioned ~1 m apart	To measure the impact of the human’s distance from the animal

**Table 2:** Sample demographic characteristics compared with the Australian population.

	Zoo Community Sample %	General Public Sample %	Australian Public ^1^ %
Gender Identity			
Male	15.4	49.8	49.6
Female	83.4	50	50.4
Non-binary	1.2	0.2	>0.01 ^a^
Age			
18–29	27.8	10.3	19.3 ^b^
30–44	35.4	30.2	27.7
45–59	22.3	27.6	25.5
60–74	13.3	24.7	19
75+	1.2	7.1	8.8
Residential Location			
Urban	86.2	87.9	89.9
Rural	13.8	12.1	10.1
Highest Level of Education			
Year 9–12	17.6	27.1	39.4
Diploma	11.6	13.8	24.6
Undergraduate	32.9	24.8	22 ^c^
Postgraduate	30.5	30.7	
Doctorate	4.8	1.7	
Zoo/Animal Sanctuary Member			
Yes	45	8.1	N/A
Member of Conservation Organisation			
Yes	25.8	7.4	N/A
Frequency of Zoo Visits			
Regularly (more than once a month) Sometimes (once every few months) Not very often (once or twice in the past 12 months) Not in the past 12 months Never	9.6 27 32.5 19.5 1.3	2.3 9.9 32.2 49.6 5.3	N/A N/A N/A N/A N/A

^1^ Australian percentages from the Australian Bureau of Statistics [53]. ^a^ “This count is not considered to be an accurate count, due to limitations around the special procedures and willingness or opportunity to report as sex and/or gender diverse. People who have been treated with disrespect, abuse and discrimination because of their sex or gender may be unwilling to reveal their sex (or gender) in an official document” [53]. ^b^ Missing data from ages 18–19 (not provided by ABS). ^c^ Only compiled data across all university qualifications were available.

**Table 3:** Test statistics and effect sizes for variables in the final model to assess respondents’ agreement with the statement “the featured animal is displaying a natural behaviour”. Parameter estimate (β), standard error (SE), Wald statistics (Wald), degrees of freedom (df), level of significance (*p*), cumulative odds ratio (Exp (β)), and 95% confidence interval of the cumulative odds ratio (95% CI Exp (β)) for variables in the model. The answer options disagree, slightly disagree, neither agree nor disagree, slightly agree, and agree were coded as 1, 2, 3, 4, 5.

								95% CI Exp(ß)	
Variable	Category	β	Std Error	Wald	df	*p*	Exp(ß)	Lower Bound	Upper Bound
Human Position (Animal alone = reference)	Touching	−1.7	0.19	74.9	1	<0.001	0.186	0.12	1.145
	~30 cm apart	−0.7	0.2	14.59	1	<0.001	0.475	0.32	1.38
	~1 m apart	−0.7	0.2	12.07	1	<0.001	0.5	0.33	1.4
Animal (Kangaroo = reference)	Parrot	−0.029	0.172	0.029	1	0.865	0.97	−0.366	0.307
	Python	0.44	0.183	5.8	1	0.016	1.55	0.082	0.799
	Stick Insect	0.774	0.195	15.717	1	<0.001	2.17	0.392	1.157

Significant results are highlighted in grey.

**Table 4:** Test statistics and effect sizes for variables in the final model to assess respondents’ agreement with the statement “the featured animal is displaying a natural behaviour”. Parameter estimate (β), standard error (SE), Wald statistics (Wald), degrees of freedom (df), level of significance (*p*), cumulative odds ratio (Exp (β)), and 95% confidence interval of the cumulative odds ratio (95% CI Exp (β)) for variables in the model. The answer options disagree, slightly disagree, neither agree nor disagree, slightly agree, and agree were coded as 1, 2, 3, 4, 5.

								95% CI Exp(ß)	
Variable	Category	β	Std Error	Wald	df	*p*	Exp(ß)	Lower Bound	Upper Bound
Human Position (Animal alone = reference)	Touching	−1.62	0.14	19.09	1	<0.001	0.54	0.41	0.71
	~30 cm apart	−0.37	0.14	6.65	1	0.01	0.475	0.52	0.91
	~1 m apart	−0.19	0.14	1.82	1	0.178	0.5	0.62	1.09
Animal (Kangaroo = reference)	Parrot	0.216	0.134	2.61	1	0.106	1.24	−0.046	0.478
	Python	0.613	0.139	19.521	1	<0.001	1.85	0.341	0.885
	Stick Insect	0.509	0.142	12.827	1	<0.001	1.66	0.23	0.787

Significant results are highlighted in grey.

**Table 5:** Test statistics and effect sizes for variables in the final model to assess respondents’ agreement with the statement “the featured animal would make a good pet”. Parameter estimate (β), standard error (SE), Wald statistics (Wald), degrees of freedom (df), level of significance (*p*), cumulative odds ratio (Exp (β)), and 95% confidence interval of the cumulative odds ratio (95% CI Exp (β)) for variables in the model. The answer options disagree, slightly disagree, neither agree nor disagree, slightly agree, and agree were coded as 1, 2, 3, 4, 5.

								95% CI Exp(β)	
Variable	Category	β	Std Error	Wald	df	*p*	Exp(β)	Lower Bound	Upper Bound
Human Position (Animal alone = reference)	Touching	0.44	0.14	10.44	1	<0.001	1.56	1.19	2.04
	~30 cm apart	0.35	0.14	6.34	1	0.01	1.42	1.08	1.86
	~1 m apart	0.25	0.14	3.28	1	0.07	1.29	0.98	1.69
Animal (Kangaroo = reference)	Parrot	1.463	0.136	115.238	1	<0.001	4.32	1.196	1.73
	Python	0.049	0.138	0.124	1	0.724	1.05	−0.221	0.318
	Stick Insect	1.027	0.141	52.802	1	<0.001	2.79	0.75	1.304

Significant results are highlighted in grey.

## Data Availability

The data presented in this study are openly available in Figshare at https://doi.org/10.6084/m9.figshare.14093303.v1 (accessed on 21 November 2021).

## Supplementary Materials

The following supporting information can be downloaded at: https://www.mdpi.com/article/10.3390/ani12010011/s1, Supplementary File 1: Online survey; Figure S1: Database of all allocated images in the study.
