# Supplementary material for: Human Positioning in Close-Encounter Photographs and the Effect on Public Perceptions of Zoo Animals
Source: Animals (Basel). 2021 Dec 21;12(1):11. doi: 10.3390/ani12010011 (PMC8749715; doi:10.3390/ani12010011)
Supplement: Supplementary file 1 [file animals-12-00011-s001.zip › Supplementary File S1.pdf]

Supplementary File S1. Online Survey.

This page of questions applies to the photo shown below. Please take a minute to examine it before answering the next question.

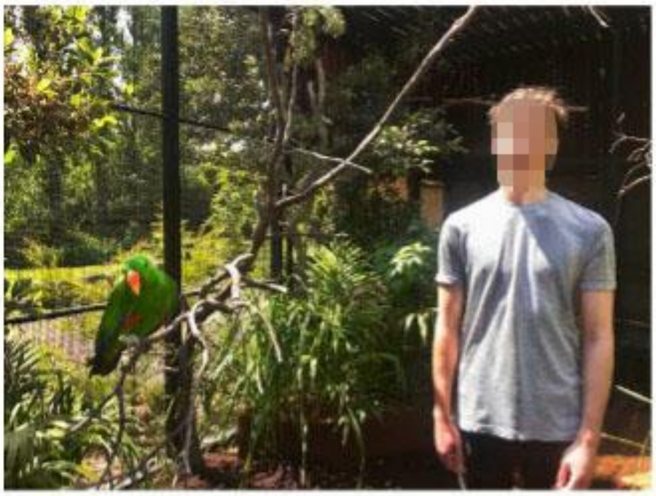

Please rate how well each of the below words describes **the zoo animal** in the above photo: *

	Not at all so	Slightly so	Moderately so	Very much so	Extremely so	Unsure
Happy	O	O	O	O	O	O
Sad	O	O	O	O	O	O
Relaxed	O	O	O	O	O	O
Nervous	O	O	O	O	O	O
Friendly	O	O	O	O	O	O
Dangerous	O	O	O	O	O	O

* This question addressed a separate research question that is not included in this thesis

How much do you agree or disagree with the following statements? *

	Strongly disagree	Disagree	Somewhat disagree	Neither agree nor disagree	Somewhat agree	Agree	Strongly agree
Zoos play an important role in the conservation of threatened species	○	○	○	○	○	○	○
Animals in a zoo are as healthy as those in the wild	○	○	○	○	○	○	○
It matters to me if an animal is in pain	○	○	○	○	○	○	○
Animals in a zoo are as happy as those in the wild	○	○	○	○	○	○	○

	Strongly disagree	Disagree	Somewhat disagree	Neither agree nor disagree	Somewhat agree	Agree	Strongly agree
Zoos are important for educating the public about animals	○	○	○	○	○	○	○
I enjoy going to the zoo	○	○	○	○	○	○	○
I feel a strong emotional bond with animals.	○	○	○	○	○	○	○
People who want to hunt should be provided the opportunity to do so	○	○	○	○	○	○	○

* These questions addressed a separate research question that is not included in this thesis

	Strongly disagree	Disagree	Somewhat disagree	Neither agree nor disagree	Somewhat agree	Agree	Strongly agree
Going to the zoo is a fun family activity	○	○	○	○	○	○	○
Humans should manage wildlife populations so that humans benefit	○	○	○	○	○	○	○
Zoos should offer opportunities for people to take photos with the animals	○	○	○	○	○	○	○
Most animals are more like computer programs, i.e. mechanically responding to instinctive urges without awareness of what they are doing	○	○	○	○	○	○	○

How much do you agree or disagree with the following statements? *

	Strongly disagree	Disagree	Somewhat disagree	Neither agree nor disagree	Somewhat agree	Agree	Strongly agree
It is acceptable for animals to be raised for human consumption	○	○	○	○	○	○	○
Animals should have rights similar to the rights of humans	○	○	○	○	○	○	○
Zoos are engaging for visitors	○	○	○	○	○	○	○
The use of animals for furs, skins and leather is acceptable	○	○	○	○	○	○	○

	Strongly disagree	Disagree	Somewhat disagree	Neither agree nor disagree	Somewhat agree	Agree	Strongly agree
Most animals are unaware of what is happening to them	○	○	○	○	○	○	○
We should strive for a world where humans and wildlife can live side by side in harmony	○	○	○	○	○	○	○
Zoos, in general, care for the wellbeing of animals	○	○	○	○	○	○	○
Zoos should offer opportunities for people to touch the animals	○	○	○	○	○	○	○

* These questions addressed a separate research question that is not included in this thesis

	Strongly disagree	Disagree	Somewhat disagree	Neither agree nor disagree	Somewhat agree	Agree	Strongly agree
The needs of humans should take priority over wildlife protection	○	○	○	○	○	○	○
It is acceptable to use wildlife in research even if it may harm some animals	○	○	○	○	○	○	○
I care about animals as much as I do other people	○	○	○	○	○	○	○

Finally, we would like to learn a little bit more about you. None of this information will be used to identify you individually, and all responses will be kept anonymous.

How did you access this survey?

- Facebook
- Instagram
- Snapchat
- Email
- Other (please specify)

Roughly how often do you visit a zoo or animal sanctuary? (select one)

- Regularly (more than once a month)
- Sometimes (once every few months)
- Not very often (once or twice in the past 12 months)
- Not in the past 12 months
- Never

Are you (or a child in your immediate family) a member of a zoo or animal sanctuary?

- Yes
- No

Are you a member of a conservation organisation?

- Yes
- No

Are you or have you been a pet owner? (select one)

- I am a pet owner currently
- I have been a pet owner in the past, but don't have one currently
- I have never been a pet owner

What is your gender identity?

- Male
- Female
- Non-Binary/Gender Non-conforming
- Prefer not to say

In what year were you born?

What is the highest level of education you have completed or are currently completing? (select one)

Year 9	Bachelor Degree
Year 10	Graduate Certificate/Diploma
Year 11	Master Degree
Year 12	Doctoral Degree
Diploma	Other (please specify)
Advanced Diploma and Associate Degree	

Do you reside in an urban or rural locality?

- Urban (within towns or cities with more than 1,000 residents)
- Rural (within a town of less than 1,000 residents or outside of a town or city)

Do you have any further comments?

As a thank you for your time, we invite you to take part in our prize draw for six $50 gift cards. To enter, please fill out the this section so that we can contact you in the event of a win. This data will be stored in a different file to your survey responses, and thus we will not be able to link you to your answers. If you do not wish to be entered in the prize draw, please skip this section and press 'continue'.

Please enter your email address if you wish to enter the prize draw

By pressing 'Continue' you are submitting your survey.

Continue
