# Supplementary figures and images for: Human Positioning in Close-Encounter Photographs and the Effect on Public Perceptions of Zoo Animals
Source: Animals (Basel). 2021 Dec 21;12(1):11. doi: 10.3390/ani12010011 (PMC8749715; doi:10.3390/ani12010011)

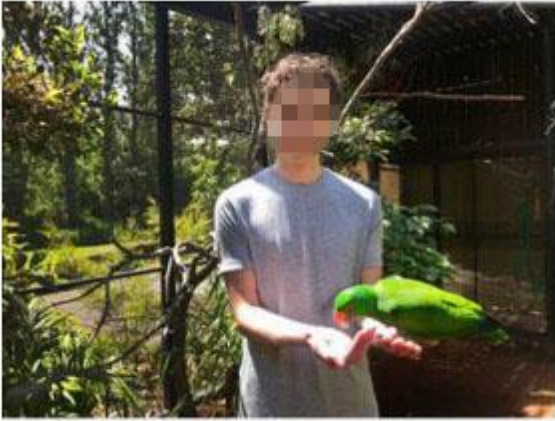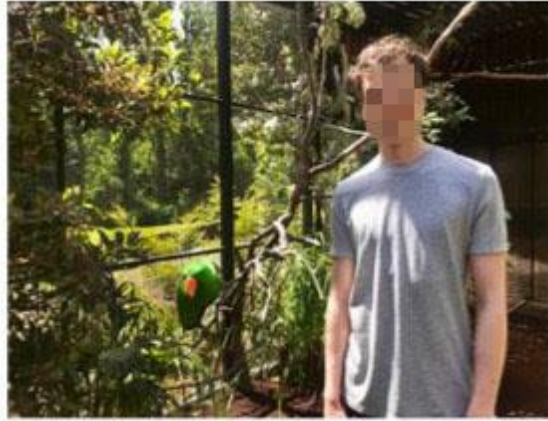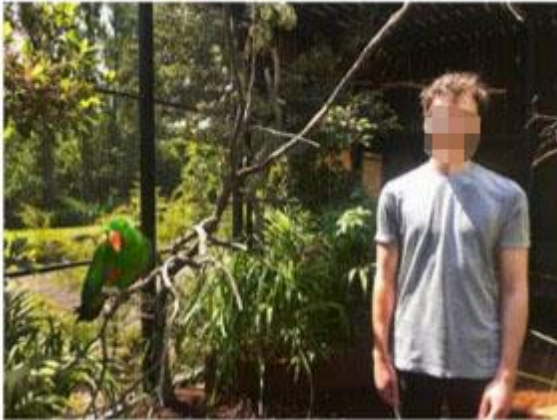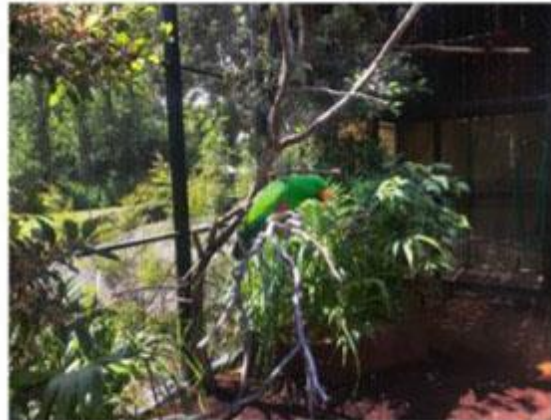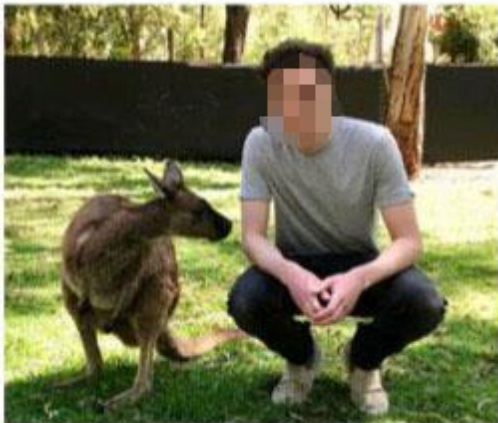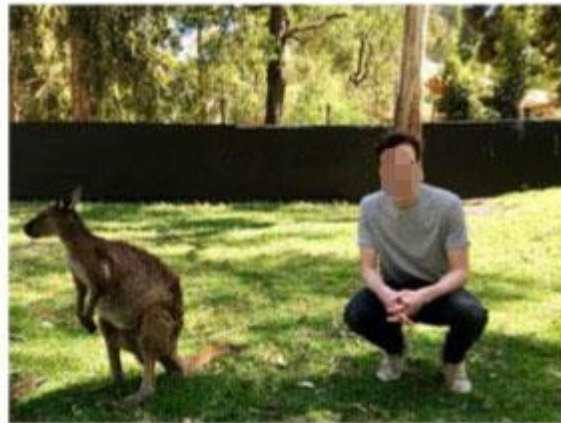

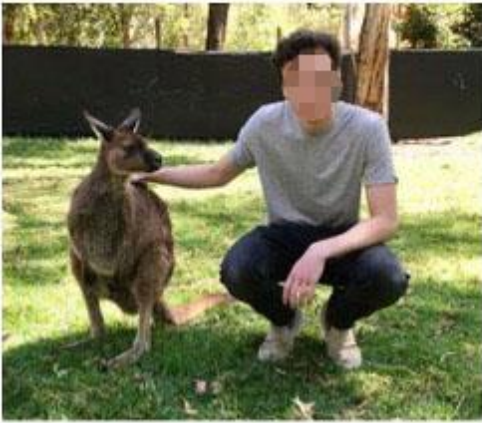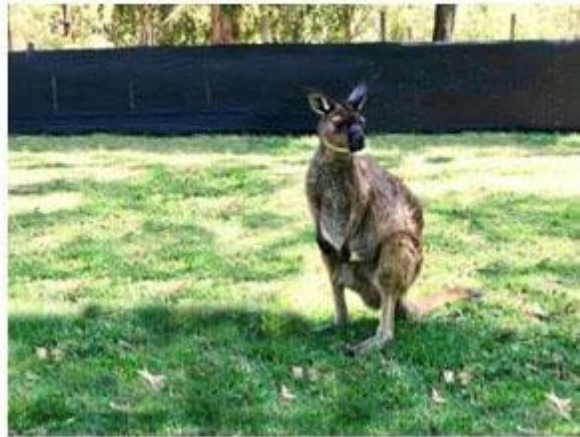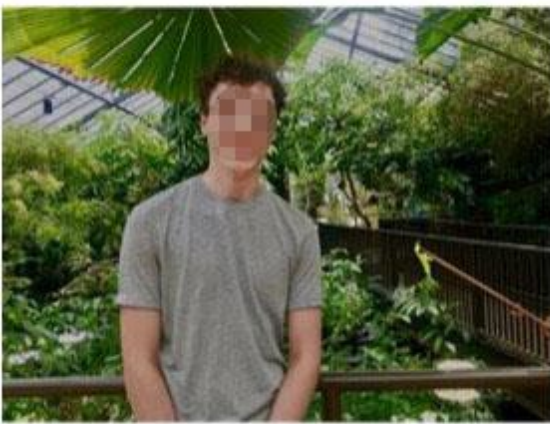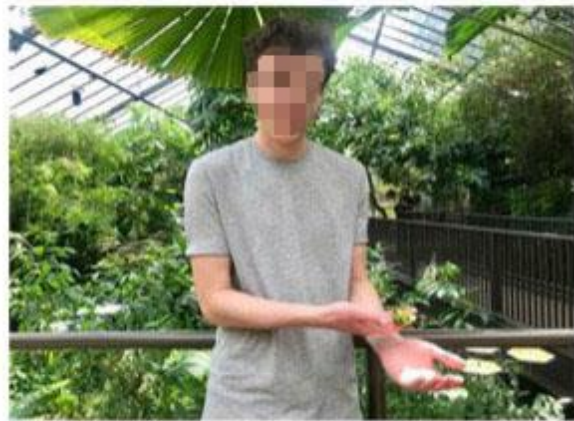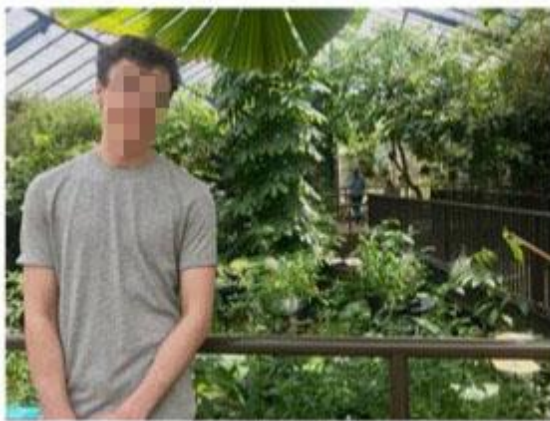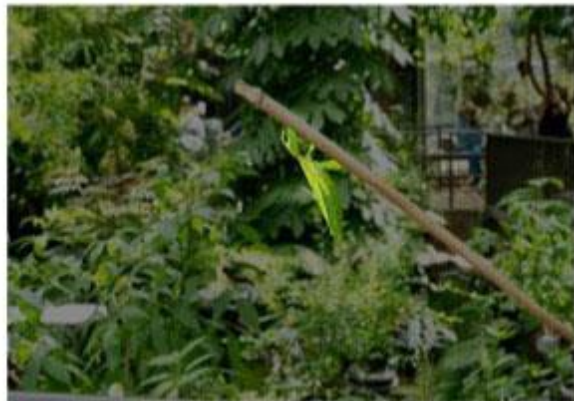

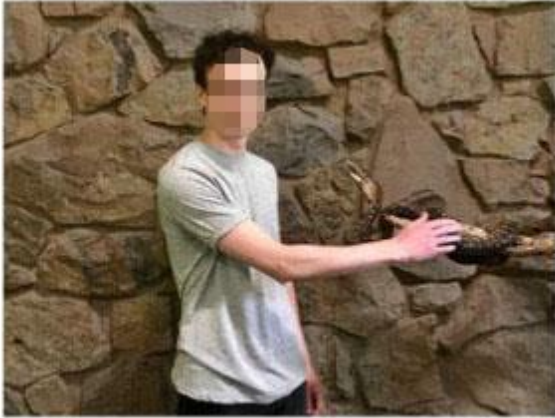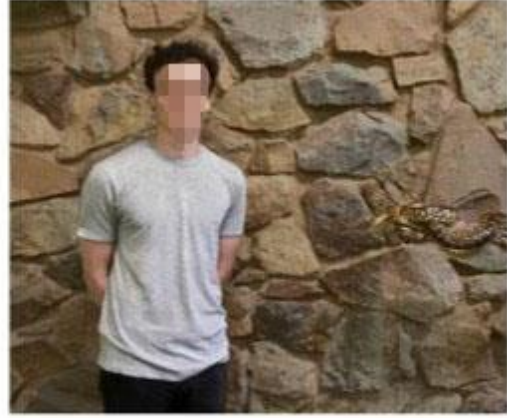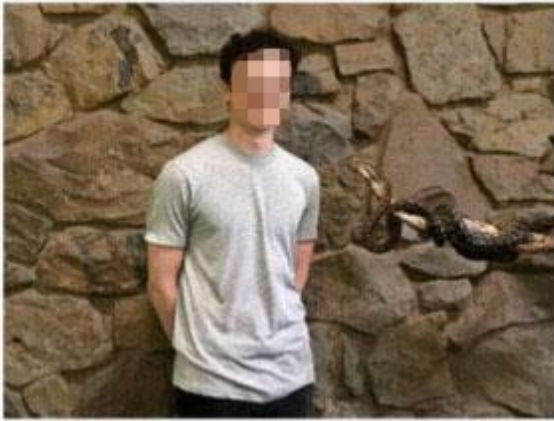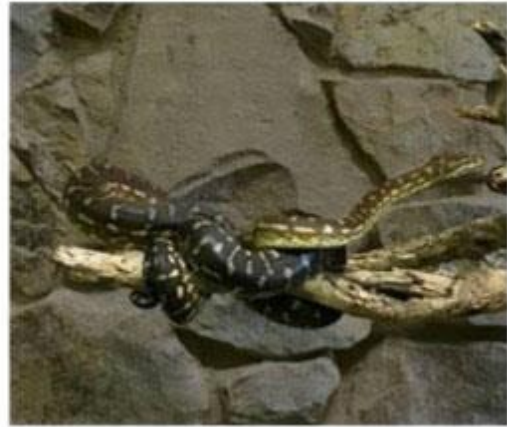

**Supplementary Figure S1.** Database of all allocated images in the study.

Supplement: Supplementary file 1 [file animals-12-00011-s001.zip › Figure S1.pdf]
